# Supplementary material for: Water quality assessment and source identification of the Shuangji River (China) using multivariate statistical methods
Source: PLoS One. 2021 Jan 22;16(1):e0245525. doi: 10.1371/journal.pone.0245525 (PMC7822302; doi:10.1371/journal.pone.0245525)
Supplement: S1 Raw data — (DOC) [file pone.0245525.s001.doc]

|  | NO. | PH | DO | COD | NH_3_-N | TP | CODMn | F | Oil | Cr^6+^ | LAS | Cu | Zn | Cd | As | Hg | CN | VP | S | Se |
| --- | --- | --- | --- | --- | --- | --- | --- | --- | --- | --- | --- | --- | --- | --- | --- | --- | --- | --- | --- | --- |
| 2019.07 | M1 | 7.47 | 6.9 | 32 | 1.48 | 0.36 | 7.5 | 0.75 | 0.24 | 0.021 | 0.29 | 0.07 | 0.05 | 0.0003 | 0.0032 | 0.00026 | 0.018 | 0.0012 | 0.012 | 0.0006 |
| 2019.07 | M2 | 7.58 | 8.5 | 16 | 1.12 | 0.24 | 4.6 | 0.76 | 0.15 | 0.013 | 0.07 | 0.06 | 0.03 | 0.0003 | 0.0034 | 0.00025 | 0.016 | 0.0006 | 0.013 | 0.0006 |
| 2019.07 | T1 | 7.49 | 8.2 | 26 | 1.24 | 0.32 | 7.1 | 0.81 | 0.19 | 0.021 | 0.15 | 0.08 | 0.05 | 0.0003 | 0.0036 | 0.00026 | 0.014 | 0.0012 | 0.014 | 0.0008 |
| 2019.07 | T2 | 7.52 | 8.3 | 15 | 0.88 | 0.15 | 5.9 | 0.39 | 0.09 | 0.023 | 0.12 | 0.04 | 0.04 | 0.0002 | 0.0014 | 0.00012 | 0.008 | 0.0007 | 0.008 | 0.0004 |
| 2019.07 | T3 | 7.79 | 8.2 | 14 | 0.82 | 0.14 | 6.1 | 0.38 | 0.08 | 0.019 | 0.14 | 0.03 | 0.06 | 0.0002 | 0.0016 | 0.0003 | 0.006 | 0.0005 | 0.009 | 0.0003 |
| 2019.07 | M3 | 7.65 | 8.4 | 23 | 1.32 | 0.21 | 4.8 | 0.62 | 0.07 | 0.021 | 0.13 | 0.05 | 0.06 | 0.0003 | 0.0011 | 0.00025 | 0.007 | 0.0009 | 0.005 | 0.0004 |
| 2019.07 | M4 | 7.31 | 8.2 | 28 | 0.77 | 0.23 | 7.1 | 0.76 | 0.07 | 0.022 | 0.15 | 0.05 | 0.04 | 0.0002 | 0.0028 | 0.00022 | 0.009 | 0.0012 | 0.011 | 0.0004 |
| 2019.07 | T4 | 7.32 | 8.2 | 19 | 0.96 | 0.21 | 4.9 | 0.65 | 0.08 | 0.015 | 0.13 | 0.04 | 0.05 | 0.0003 | 0.0022 | 0.00015 | 0.011 | 0.0006 | 0.006 | 0.0005 |
| 2019.07 | T5 | 7.32 | 7.9 | 18 | 0.82 | 0.11 | 3.1 | 0.62 | 0.06 | 0.015 | 0.14 | 0.05 | 0.03 | 0.0002 | 0.002 | 0.00016 | 0.01 | 0.0005 | 0.005 | 0.0004 |
| 2019.07 | M5 | 7.28 | 7.8 | 36 | 0.92 | 0.33 | 8.1 | 0.75 | 0.07 | 0.026 | 0.13 | 0.06 | 0.04 | 0.0005 | 0.0028 | 0.00018 | 0.018 | 0.0008 | 0.006 | 0.0008 |
| 2019.07 | M6 | 7.32 | 7.6 | 38 | 0.86 | 0.19 | 7.8 | 0.73 | 0.08 | 0.022 | 0.11 | 0.05 | 0.05 | 0.0005 | 0.0025 | 0.00016 | 0.017 | 0.001 | 0.005 | 0.0006 |
| 2019.07 | T6 | 7.32 | 8.2 | 32 | 1.21 | 0.31 | 7.6 | 0.72 | 0.06 | 0.023 | 0.2 | 0.06 | 0.05 | 0.0004 | 0.0032 | 0.00018 | 0.008 | 0.0009 | 0.008 | 0.0008 |
| 2019.07 | M7 | 7.42 | 8.3 | 29 | 0.75 | 0.28 | 7.5 | 0.71 | 0.07 | 0.023 | 0.16 | 0.05 | 0.04 | 0.0004 | 0.0028 | 0.00016 | 0.015 | 0.0009 | 0.006 | 0.0007 |
| 2019.07 | M8 | 7.42 | 8.8 | 23 | 0.68 | 0.26 | 7.2 | 0.68 | 0.06 | 0.022 | 0.17 | 0.05 | 0.03 | 0.0003 | 0.0026 | 0.00021 | 0.011 | 0.0008 | 0.005 | 0.0007 |
| 2019.08 | M1 | 7.46 | 6.8 | 33 | 1.47 | 0.38 | 7.4 | 0.74 | 0.23 | 0.020 | 0.3 | 0.07 | 0.04 | 0.0003 | 0.0032 | 0.00026 | 0.018 | 0.0012 | 0.012 | 0.0006 |
| 2019.08 | M2 | 7.88 | 8.7 | 18 | 1.02 | 0.21 | 4.8 | 0.78 | 0.14 | 0.012 | 0.28 | 0.06 | 0.03 | 0.0003 | 0.0034 | 0.00025 | 0.016 | 0.0006 | 0.013 | 0.0006 |
| 2019.08 | T1 | 7.46 | 8.9 | 28 | 1.28 | 0.36 | 7.2 | 0.82 | 0.18 | 0.024 | 0.14 | 0.08 | 0.05 | 0.0003 | 0.0036 | 0.00026 | 0.014 | 0.0012 | 0.014 | 0.0008 |
| 2019.08 | T2 | 7.57 | 8.2 | 16 | 0.86 | 0.14 | 5.8 | 0.38 | 0.08 | 0.022 | 0.11 | 0.04 | 0.03 | 0.0002 | 0.0014 | 0.00012 | 0.008 | 0.0007 | 0.008 | 0.0004 |
| 2019.08 | T3 | 7.77 | 8.7 | 15 | 0.84 | 0.13 | 6.0 | 0.41 | 0.09 | 0.018 | 0.13 | 0.03 | 0.05 | 0.0002 | 0.0016 | 0.0003 | 0.006 | 0.0005 | 0.009 | 0.0003 |
| 2019.08 | M3 | 7.67 | 8.6 | 24 | 1.29 | 0.2 | 4.6 | 0.61 | 0.06 | 0.019 | 0.12 | 0.05 | 0.06 | 0.0003 | 0.0011 | 0.00026 | 0.007 | 0.0009 | 0.005 | 0.0004 |
| 2019.08 | M4 | 7.32 | 8.3 | 28 | 0.81 | 0.22 | 7 | 0.77 | 0.06 | 0.021 | 0.14 | 0.05 | 0.06 | 0.0002 | 0.0028 | 0.00022 | 0.009 | 0.0012 | 0.01 | 0.0004 |
| 2019.08 | T4 | 7.33 | 8.8 | 18 | 0.97 | 0.22 | 4.7 | 0.66 | 0.08 | 0.014 | 0.13 | 0.04 | 0.05 | 0.0003 | 0.0022 | 0.00013 | 0.011 | 0.0006 | 0.006 | 0.0005 |
| 2019.08 | T5 | 7.35 | 8.8 | 19 | 0.84 | 0.12 | 3 | 0.61 | 0.06 | 0.014 | 0.15 | 0.05 | 0.03 | 0.0002 | 0.002 | 0.00015 | 0.01 | 0.0005 | 0.005 | 0.0004 |
| 2019.08 | M5 | 7.34 | 7.5 | 44 | 0.97 | 0.32 | 8.2 | 0.76 | 0.08 | 0.024 | 0.14 | 0.06 | 0.04 | 0.0005 | 0.0028 | 0.00018 | 0.018 | 0.0008 | 0.006 | 0.0008 |
| 2019.08 | M6 | 7.33 | 7.9 | 42 | 0.85 | 0.18 | 8.0 | 0.74 | 0.06 | 0.021 | 0.12 | 0.05 | 0.05 | 0.0005 | 0.0025 | 0.00016 | 0.017 | 0.001 | 0.005 | 0.0006 |
| 2019.08 | T6 | 7.35 | 8.6 | 36 | 1.24 | 0.32 | 7.7 | 0.76 | 0.08 | 0.021 | 0.22 | 0.06 | 0.05 | 0.0004 | 0.0032 | 0.00018 | 0.008 | 0.0009 | 0.008 | 0.0008 |
| 2019.08 | M7 | 7.41 | 8.7 | 30 | 0.74 | 0.26 | 7.6 | 0.71 | 0.06 | 0.021 | 0.16 | 0.05 | 0.04 | 0.0004 | 0.0028 | 0.00016 | 0.015 | 0.0009 | 0.006 | 0.0007 |
| 2019.08 | M8 | 7.46 | 9.1 | 24 | 0.66 | 0.24 | 7.3 | 0.66 | 0.06 | 0.021 | 0.18 | 0.05 | 0.03 | 0.0003 | 0.0026 | 0.00015 | 0.011 | 0.0008 | 0.005 | 0.0007 |
| 2019.09 | M1 | 7.43 | 8.2 | 33 | 1.43 | 0.36 | 8.3 | 0.66 | 0.18 | 0.023 | 0.26 | 0.06 | 0.06 | 0.0002 | 0.0026 | 0.00015 | 0.016 | 0.001 | 0.012 | 0.0013 |
| 2019.09 | M2 | 7.31 | 8.8 | 13 | 1.68 | 0.32 | 3.5 | 0.94 | 0.09 | 0.012 | 0.22 | 0.05 | 0.05 | 0.0002 | 0.002 | 0.00019 | 0.015 | 0.0004 | 0.007 | 0.0009 |
| 2019.09 | T1 | 7.46 | 8.7 | 28 | 1.23 | 0.37 | 7.8 | 1.1 | 0.16 | 0.020 | 0.11 | 0.08 | 0.06 | 0.0003 | 0.0026 | 0.00016 | 0.012 | 0.0009 | 0.014 | 0.0012 |
| 2019.09 | T2 | 7.27 | 7.8 | 10 | 1.42 | 0.14 | 5 | 0.46 | 0.08 | 0.010 | 0.09 | 0.04 | 0.05 | 0.0002 | 0.0028 | 0.00019 | 0.007 | 0.0005 | 0.008 | 0.0007 |
| 2019.09 | T3 | 7.28 | 9.2 | 10 | 1.76 | 0.14 | 5.1 | 0.38 | 0.06 | 0.010 | 0.08 | 0.03 | 0.04 | 0.0002 | 0.0022 | 0.00019 | 0.006 | 0.0004 | 0.01 | 0.0006 |
| 2019.09 | M3 | 7.32 | 9.5 | 15 | 1.27 | 0.12 | 6.3 | 0.59 | 0.06 | 0.013 | 0.07 | 0.04 | 0.04 | 0.0003 | 0.0038 | 0.00018 | 0.006 | 0.0006 | 0.005 | 0.0008 |
| 2019.09 | M4 | 7.38 | 8.8 | 24 | 1.54 | 0.27 | 7.4 | 0.61 | 0.12 | 0.020 | 0.09 | 0.04 | 0.05 | 0.0002 | 0.0036 | 0.00026 | 0.008 | 0.0009 | 0.011 | 0.0013 |
| 2019.09 | T4 | 7.34 | 8.9 | 9 | 0.72 | 0.21 | 4.6 | 0.71 | 0.11 | 0.010 | 0.05 | 0.03 | 0.06 | 0.0003 | 0.0032 | 0.00009 | 0.01 | 0.0004 | 0.006 | 0.0006 |
| 2019.09 | T5 | 7.29 | 8.4 | 15 | 0.76 | 0.11 | 2.5 | 0.63 | 0.02 | 0.012 | 0.04 | 0.03 | 0.07 | 0.0002 | 0.0026 | 0.00008 | 0.009 | 0.0003 | 0.005 | 0.0008 |
| 2019.09 | M5 | 7.74 | 9.6 | 37 | 0.95 | 0.2 | 7.2 | 0.69 | 0.06 | 0.022 | 0.08 | 0.04 | 0.05 | 0.0004 | 0.0035 | 0.00016 | 0.017 | 0.0006 | 0.006 | 0.0013 |
| 2019.09 | M6 | 7.36 | 9.0 | 24 | 0.74 | 0.16 | 7.5 | 0.9 | 0.14 | 0.020 | 0.07 | 0.03 | 0.06 | 0.0004 | 0.0032 | 0.00013 | 0.015 | 0.0008 | 0.006 | 0.0008 |
| 2019.09 | T6 | 7.42 | 8.6 | 32 | 1.42 | 0.18 | 8.5 | 0.98 | 0.16 | 0.024 | 0.08 | 0.04 | 0.06 | 0.0003 | 0.0036 | 0.00015 | 0.006 | 0.0007 | 0.009 | 0.0012 |
| 2019.09 | M7 | 7.38 | 9.1 | 26 | 1.21 | 0.17 | 7.8 | 0.84 | 0.13 | 0.021 | 0.07 | 0.03 | 0.05 | 0.0003 | 0.0032 | 0.00012 | 0.013 | 0.0007 | 0.007 | 0.001 |
| 2019.09 | M8 | 7.41 | 9.3 | 29 | 0.62 | 0.15 | 8.3 | 0.72 | 0.12 | 0.020 | 0.06 | 0.02 | 0.04 | 0.0003 | 0.003 | 0.00009 | 0.009 | 0.0006 | 0.006 | 0.0009 |
| 2019.10 | M1 | 7.45 | 6.8 | 30 | 1.02 | 0.44 | 8.4 | 0.62 | 0.15 | 0.022 | 0.16 | 0.05 | 0.05 | 0.0003 | 0.0022 | 0.0001 | 0.017 | 0.0015 | 0.011 | 0.001 |
| 2019.10 | M2 | 7.52 | 8.4 | 9 | 0.42 | 0.19 | 3.6 | 0.91 | 0.07 | 0.009 | 0.14 | 0.04 | 0.04 | 0.0003 | 0.0012 | 0.00014 | 0.016 | 0.0009 | 0.009 | 0.0005 |
| 2019.10 | T1 | 7.88 | 8.7 | 25 | 1.24 | 0.24 | 7.8 | 0.94 | 0.16 | 0.018 | 0.13 | 0.07 | 0.04 | 0.0003 | 0.002 | 0.00011 | 0.015 | 0.0012 | 0.014 | 0.0009 |
| 2019.10 | T2 | 7.83 | 8.2 | 4 | 1.32 | 0.12 | 5.1 | 0.42 | 0.08 | 0.007 | 0.12 | 0.03 | 0.03 | 0.0002 | 0.0008 | 0.00011 | 0.009 | 0.0007 | 0.006 | 0.0005 |
| 2019.10 | T3 | 7.57 | 8.5 | 5 | 1.63 | 0.2 | 5.2 | 0.38 | 0.07 | 0.008 | 0.12 | 0.02 | 0.02 | 0.0002 | 0.0008 | 0.00013 | 0.005 | 0.0006 | 0.008 | 0.0004 |
| 2019.10 | M3 | 7.95 | 8.6 | 6 | 1.19 | 0.28 | 6.5 | 0.52 | 0.06 | 0.013 | 0.16 | 0.03 | 0.03 | 0.0004 | 0.0012 | 0.00014 | 0.008 | 0.0012 | 0.009 | 0.0006 |
| 2019.10 | M4 | 7.85 | 8.1 | 25 | 1.78 | 0.32 | 7.5 | 0.58 | 0.12 | 0.019 | 0.18 | 0.03 | 0.05 | 0.0003 | 0.002 | 0.00016 | 0.01 | 0.0016 | 0.012 | 0.0008 |
| 2019.10 | T4 | 7.65 | 8.8 | 6 | 0.44 | 0.23 | 4.5 | 0.7 | 0.07 | 0.012 | 0.09 | 0.02 | 0.04 | 0.0003 | 0.0009 | 0.00006 | 0.012 | 0.0009 | 0.008 | 0.0005 |
| 2019.10 | T5 | 7.83 | 8.5 | 24 | 0.67 | 0.14 | 2.4 | 0.6 | 0.04 | 0.008 | 0.08 | 0.02 | 0.03 | 0.0001 | 0.0012 | 0.00005 | 0.01 | 0.0008 | 0.009 | 0.0005 |
| 2019.10 | M5 | 7.92 | 7.1 | 31 | 0.83 | 0.13 | 7.3 | 0.63 | 0.12 | 0.020 | 0.16 | 0.03 | 0.06 | 0.0003 | 0.0023 | 0.00015 | 0.018 | 0.0013 | 0.008 | 0.0004 |
| 2019.10 | M6 | 7.71 | 7.6 | 22 | 0.72 | 0.12 | 7.1 | 0.84 | 0.18 | 0.018 | 0.15 | 0.02 | 0.05 | 0.0002 | 0.0021 | 0.00007 | 0.018 | 0.0012 | 0.007 | 0.0006 |
| 2019.10 | T6 | 7.75 | 8.3 | 9 | 0.73 | 0.22 | 8.2 | 0.96 | 0.16 | 0.021 | 0.16 | 0.03 | 0.05 | 0.0003 | 0.0022 | 0.00008 | 0.009 | 0.0009 | 0.01 | 0.0005 |
| 2019.10 | M7 | 7.85 | 8.4 | 25 | 0.79 | 0.09 | 7.8 | 0.82 | 0.17 | 0.019 | 0.15 | 0.02 | 0.05 | 0.0002 | 0.002 | 0.00007 | 0.016 | 0.0012 | 0.008 | 0.0004 |
| 2019.10 | M8 | 7.95 | 8.8 | 19 | 0.45 | 0.15 | 7.2 | 0.69 | 0.16 | 0.018 | 0.14 | 0.02 | 0.04 | 0.0002 | 0.0018 | 0.00006 | 0.012 | 0.001 | 0.007 | 0.0004 |
| 2019.11 | M1 | 7.81 | 8.1 | 26 | 1.43 | 0.43 | 7.5 | 0.46 | 0.19 | 0.019 | 0.29 | 0.06 | 0.06 | 0.0002 | 0.0016 | 0.00013 | 0.013 | 0.001 | 0.01 | 0.0009 |
| 2019.11 | M2 | 7.62 | 9.1 | 10 | 1.47 | 0.23 | 2.8 | 0.72 | 0.18 | 0.012 | 0.28 | 0.05 | 0.04 | 0.0002 | 0.0008 | 0.00009 | 0.009 | 0.0003 | 0.008 | 0.0004 |
| 2019.11 | T1 | 7.49 | 8.2 | 26 | 1.28 | 0.28 | 8.2 | 0.78 | 0.2 | 0.018 | 0.26 | 0.08 | 0.04 | 0.0003 | 0.0015 | 0.00012 | 0.012 | 0.0009 | 0.011 | 0.0006 |
| 2019.11 | T2 | 7.48 | 8.6 | 6 | 1.25 | 0.12 | 5.2 | 0.43 | 0.05 | 0.008 | 0.22 | 0.04 | 0.04 | 0.0003 | 0.0009 | 0.00009 | 0.007 | 0.0003 | 0.007 | 0.0005 |
| 2019.11 | T3 | 7.65 | 8.8 | 6 | 1.31 | 0.08 | 5.8 | 0.42 | 0.05 | 0.008 | 0.23 | 0.03 | 0.03 | 0.0003 | 0.0009 | 0.00018 | 0.006 | 0.0003 | 0.006 | 0.0004 |
| 2019.11 | M3 | 7.75 | 8.2 | 15 | 1.13 | 0.33 | 6.6 | 0.61 | 0.05 | 0.015 | 0.18 | 0.04 | 0.05 | 0.0003 | 0.0013 | 0.00015 | 0.014 | 0.0004 | 0.008 | 0.0006 |
| 2019.11 | M4 | 7.78 | 8.4 | 21 | 1.40 | 0.27 | 5.4 | 0.63 | 0.17 | 0.018 | 0.26 | 0.05 | 0.03 | 0.0003 | 0.0015 | 0.00013 | 0.013 | 0.0008 | 0.011 | 0.0009 |
| 2019.11 | T4 | 7.77 | 7.9 | 5 | 0.41 | 0.23 | 2.5 | 0.51 | 0.06 | 0.016 | 0.08 | 0.03 | 0.04 | 0.0001 | 0.0008 | 0.00009 | 0.006 | 0.0004 | 0.006 | 0.0005 |
| 2019.11 | T5 | 7.35 | 8.0 | 6 | 0.39 | 0.13 | 2.4 | 0.55 | 0.06 | 0.006 | 0.12 | 0.02 | 0.05 | 0.0003 | 0.0008 | 0.00008 | 0.005 | 0.0003 | 0.005 | 0.0004 |
| 2019.11 | M5 | 7.9 | 8 | 15 | 0.67 | 0.17 | 6.6 | 0.65 | 0.06 | 0.020 | 0.09 | 0.04 | 0.03 | 0.0004 | 0.0012 | 0.00015 | 0.012 | 0.0006 | 0.008 | 0.0006 |
| 2019.11 | M6 | 7.62 | 8.3 | 13 | 0.57 | 0.1 | 4.5 | 0.79 | 0.13 | 0.020 | 0.27 | 0.03 | 0.04 | 0.0003 | 0.0009 | 0.00013 | 0.008 | 0.0004 | 0.007 | 0.0004 |
| 2019.11 | T6 | 7.62 | 8.6 | 22 | 0.46 | 0.17 | 6.3 | 0.86 | 0.15 | 0.018 | 0.26 | 0.04 | 0.03 | 0.0003 | 0.0012 | 0.00015 | 0.009 | 0.0005 | 0.006 | 0.0005 |
| 2019.11 | M7 | 7.48 | 8.2 | 16 | 0.64 | 0.10 | 6.1 | 0.83 | 0.1 | 0.017 | 0.28 | 0.03 | 0.04 | 0.0002 | 0.0008 | 0.00012 | 0.006 | 0.0004 | 0.006 | 0.0004 |
| 2019.11 | M8 | 7.49 | 9.18 | 20 | 0.74 | 0.12 | 5.2 | 0.85 | 0.13 | 0.016 | 0.26 | 0.02 | 0.03 | 0.0002 | 0.0007 | 0.00009 | 0.004 | 0.0003 | 0.005 | 0.0004 |
| 2019.12 | M1 | 7.37 | 8.3 | 23 | 1.59 | 0.44 | 8.3 | 0.66 | 0.17 | 0.017 | 0.29 | 0.04 | 0.05 | 0.0002 | 0.0022 | 0.00013 | 0.013 | 0.0009 | 0.011 | 0.0012 |
| 2019.12 | M2 | 8.31 | 8.7 | 6 | 0.52 | 0.21 | 3.5 | 0.94 | 0.12 | 0.011 | 0.26 | 0.03 | 0.04 | 0.0001 | 0.0012 | 0.00008 | 0.005 | 0.0003 | 0.006 | 0.0006 |
| 2019.12 | T1 | 7.4 | 9 | 22 | 1.63 | 0.27 | 7.8 | 1.1 | 0.05 | 0.015 | 0.24 | 0.06 | 0.04 | 0.0002 | 0.002 | 0.00012 | 0.012 | 0.0008 | 0.009 | 0.0011 |
| 2019.12 | T2 | 7.54 | 7.7 | 11 | 1.60 | 0.08 | 5.2 | 0.46 | 0.09 | 0.012 | 0.18 | 0.04 | 0.03 | 0.0002 | 0.0013 | 0.00009 | 0.006 | 0.0003 | 0.006 | 0.0007 |
| 2019.12 | T3 | 8.05 | 9.2 | 5 | 1.49 | 0.08 | 6.1 | 0.38 | 0.08 | 0.015 | 0.17 | 0.02 | 0.03 | 0.0002 | 0.0012 | 0.00016 | 0.005 | 0.0003 | 0.005 | 0.0006 |
| 2019.12 | M3 | 7.88 | 9.6 | 16 | 0.79 | 0.36 | 6.3 | 0.59 | 0.06 | 0.012 | 0.16 | 0.03 | 0.03 | 0.0003 | 0.0019 | 0.00009 | 0.009 | 0.0005 | 0.006 | 0.0008 |
| 2019.12 | M4 | 7.98 | 8.9 | 24 | 0.86 | 0.12 | 7.4 | 0.61 | 0.08 | 0.015 | 0.21 | 0.03 | 0.04 | 0.0004 | 0.0022 | 0.00012 | 0.012 | 0.0008 | 0.009 | 0.0013 |
| 2019.12 | T4 | 7.98 | 8.7 | 16 | 0.69 | 0.16 | 4.6 | 0.71 | 0.11 | 0.018 | 0.18 | 0.03 | 0.03 | 0.0003 | 0.0018 | 0.00008 | 0.005 | 0.0005 | 0.006 | 0.0007 |
| 2019.12 | T5 | 7.93 | 8.6 | 8 | 0.49 | 0.12 | 3.5 | 0.63 | 0.02 | 0.006 | 0.16 | 0.02 | 0.05 | 0.0002 | 0.0016 | 0.00006 | 0.004 | 0.0003 | 0.005 | 0.0006 |
| 2019.12 | M5 | 7.9 | 9.7 | 33 | 0.56 | 0.12 | 7.2 | 0.69 | 0.08 | 0.017 | 0.2 | 0.04 | 0.05 | 0.0003 | 0.0025 | 0.00012 | 0.007 | 0.0004 | 0.008 | 0.0012 |
| 2019.12 | M6 | 7.71 | 9.1 | 26 | 0.52 | 0.12 | 7.5 | 0.9 | 0.14 | 0.016 | 0.18 | 0.03 | 0.03 | 0.0003 | 0.0022 | 0.0001 | 0.005 | 0.0003 | 0.006 | 0.0009 |
| 2019.12 | T6 | 7.81 | 8.8 | 13 | 1.14 | 0.26 | 8.5 | 0.98 | 0.16 | 0.017 | 0.20 | 0.04 | 0.04 | 0.0003 | 0.0023 | 0.00009 | 0.006 | 0.0003 | 0.007 | 0.0011 |
| 2019.12 | M7 | 7.8 | 8.7 | 24 | 0.44 | 0.09 | 7.8 | 0.84 | 0.13 | 0.015 | 0.19 | 0.03 | 0.03 | 0.0002 | 0.0021 | 0.00007 | 0.005 | 0.0004 | 0.006 | 0.0009 |
| 2019.12 | M8 | 7.9 | 9.2 | 20 | 0.68 | 0.08 | 8.3 | 0.72 | 0.12 | 0.015 | 0.18 | 0.02 | 0.03 | 0.0002 | 0.0019 | 0.00005 | 0.004 | 0.0003 | 0.005 | 0.0008 |
| 2020.01 | M1 | 7.43 | 7.1 | 25 | 1.74 | 0.42 | 7.7 | 0.44 | 0.15 | 0.013 | 0.26 | 0.05 | 0.06 | 0.0002 | 0.0032 | 0.00013 | 0.012 | 0.0008 | 0.011 | 0.0011 |
| 2020.01 | M2 | 7.5 | 8.6 | 8 | 0.62 | 0.19 | 2.6 | 0.87 | 0.07 | 0.008 | 0.28 | 0.04 | 0.05 | 0.0002 | 0.0018 | 0.00007 | 0.006 | 0.0004 | 0.006 | 0.0006 |
| 2020.01 | T1 | 7.88 | 8.8 | 6 | 1.52 | 0.32 | 7.2 | 0.76 | 0.05 | 0.019 | 0.28 | 0.06 | 0.05 | 0.0002 | 0.0018 | 0.00006 | 0.011 | 0.0006 | 0.009 | 0.0009 |
| 2020.01 | T2 | 7.81 | 8.1 | 11 | 1.63 | 0.12 | 3.4 | 0.45 | 0.08 | 0.014 | 0.18 | 0.03 | 0.06 | 0.0003 | 0.0018 | 0.00009 | 0.007 | 0.0005 | 0.007 | 0.0007 |
| 2020.01 | T3 | 7.46 | 8.6 | 7 | 1.31 | 0.05 | 5.5 | 0.52 | 0.07 | 0.016 | 0.16 | 0.02 | 0.04 | 0.0002 | 0.0016 | 0.00018 | 0.006 | 0.0003 | 0.006 | 0.0006 |
| 2020.01 | M3 | 7.93 | 8.5 | 10 | 1.11 | 0.13 | 6.2 | 0.61 | 0.06 | 0.013 | 0.22 | 0.03 | 0.03 | 0.0003 | 0.0026 | 0.00011 | 0.009 | 0.0006 | 0.008 | 0.0011 |
| 2020.01 | M4 | 7.84 | 8.2 | 15 | 0.62 | 0.16 | 5.2 | 0.63 | 0.08 | 0.016 | 0.26 | 0.03 | 0.03 | 0.0004 | 0.0028 | 0.00012 | 0.012 | 0.0009 | 0.01 | 0.0015 |
| 2020.01 | T4 | 7.64 | 8.9 | 8 | 0.33 | 0.21 | 2.6 | 0.53 | 0.07 | 0.013 | 0.2 | 0.02 | 0.04 | 0.0004 | 0.0018 | 0.00008 | 0.007 | 0.0005 | 0.005 | 0.0009 |
| 2020.01 | T5 | 7.82 | 8.7 | 6 | 0.58 | 0.05 | 3.6 | 0.58 | 0.04 | 0.006 | 0.18 | 0.02 | 0.04 | 0.0002 | 0.0016 | 0.00006 | 0.006 | 0.0003 | 0.005 | 0.0006 |
| 2020.01 | M5 | 7.91 | 7.3 | 14 | 0.58 | 0.11 | 3.9 | 0.66 | 0.1 | 0.012 | 0.26 | 0.04 | 0.03 | 0.0003 | 0.0036 | 0.00013 | 0.01 | 0.0005 | 0.007 | 0.0012 |
| 2020.01 | M6 | 7.69 | 7.8 | 17 | 0.96 | 0.16 | 4.9 | 0.81 | 0.18 | 0.021 | 0.26 | 0.03 | 0.03 | 0.0003 | 0.0032 | 0.00011 | 0.008 | 0.0004 | 0.006 | 0.0009 |
| 2020.01 | T6 | 7.72 | 8.5 | 11 | 0.81 | 0.2 | 5.5 | 0.88 | 0.16 | 0.018 | 0.25 | 0.04 | 0.04 | 0.0003 | 0.0034 | 0.00013 | 0.007 | 0.0006 | 0.008 | 0.0011 |
| 2020.01 | M7 | 7.86 | 8.6 | 17 | 0.45 | 0.07 | 5.3 | 0.84 | 0.17 | 0.017 | 0.24 | 0.02 | 0.03 | 0.0003 | 0.003 | 0.00011 | 0.007 | 0.0005 | 0.007 | 0.0008 |
| 2020.01 | M8 | 7.94 | 9.3 | 15 | 0.62 | 0.1 | 5.2 | 0.85 | 0.16 | 0.015 | 0.24 | 0.02 | 0.04 | 0.0002 | 0.0028 | 0.00009 | 0.006 | 0.0004 | 0.006 | 0.0007 |
| 2020.03 | M1 | 7.46 | 7.2 | 36 | 2.22 | 0.36 | 9.9 | 0.46 | 0.13 | 0.014 | 0.26 | 0.05 | 0.04 | 0.0002 | 0.0036 | 0.00013 | 0.013 | 0.0009 | 0.012 | 0.0012 |
| 2020.03 | M2 | 7.53 | 8.7 | 32 | 0.77 | 0.16 | 3.6 | 0.85 | 0.06 | 0.009 | 0.27 | 0.05 | 0.03 | 0.0002 | 0.0019 | 0.00008 | 0.007 | 0.0006 | 0.007 | 0.0008 |
| 2020.03 | T1 | 7.92 | 8.9 | 30 | 1.48 | 0.34 | 6.3 | 0.77 | 0.05 | 0.018 | 0.29 | 0.07 | 0.04 | 0.0002 | 0.0019 | 0.00006 | 0.012 | 0.0008 | 0.009 | 0.0009 |
| 2020.03 | T2 | 7.83 | 8.2 | 6 | 1.50 | 0.15 | 3.7 | 0.46 | 0.09 | 0.015 | 0.19 | 0.04 | 0.05 | 0.0003 | 0.0021 | 0.00008 | 0.008 | 0.0006 | 0.008 | 0.0008 |
| 2020.03 | T3 | 7.48 | 8.6 | 8 | 1.89 | 0.09 | 5.9 | 0.51 | 0.06 | 0.017 | 0.17 | 0.03 | 0.05 | 0.0002 | 0.0018 | 0.00017 | 0.007 | 0.0004 | 0.007 | 0.0007 |
| 2020.03 | M3 | 7.96 | 8.7 | 22 | 1.35 | 0.21 | 6.8 | 0.63 | 0.05 | 0.014 | 0.21 | 0.03 | 0.05 | 0.0003 | 0.0028 | 0.00012 | 0.009 | 0.0007 | 0.009 | 0.0012 |
| 2020.03 | M4 | 7.86 | 8.3 | 31 | 1.32 | 0.21 | 5.1 | 0.62 | 0.07 | 0.018 | 0.26 | 0.03 | 0.04 | 0.0004 | 0.0031 | 0.00013 | 0.013 | 0.0009 | 0.012 | 0.0016 |
| 2020.03 | T4 | 7.67 | 8.8 | 6 | 0.61 | 0.15 | 2.6 | 0.54 | 0.06 | 0.013 | 0.21 | 0.02 | 0.03 | 0.0004 | 0.0019 | 0.00009 | 0.008 | 0.0006 | 0.006 | 0.0009 |
| 2020.03 | T5 | 7.85 | 8.8 | 12 | 0.67 | 0.03 | 3.6 | 0.56 | 0.05 | 0.007 | 0.18 | 0.03 | 0.04 | 0.0002 | 0.0017 | 0.00007 | 0.007 | 0.0004 | 0.006 | 0.0008 |
| 2020.03 | M5 | 7.95 | 7.4 | 29 | 1.21 | 0.15 | 6.7 | 0.67 | 0.09 | 0.013 | 0.25 | 0.05 | 0.04 | 0.0003 | 0.0036 | 0.00013 | 0.012 | 0.0004 | 0.008 | 0.0013 |
| 2020.03 | M6 | 7.72 | 7.9 | 20 | 1.51 | 0.12 | 3.9 | 0.76 | 0.17 | 0.022 | 0.26 | 0.04 | 0.04 | 0.0003 | 0.0035 | 0.00012 | 0.009 | 0.0005 | 0.007 | 0.0009 |
| 2020.03 | T6 | 7.75 | 8.7 | 30 | 0.79 | 0.21 | 5.2 | 0.86 | 0.17 | 0.019 | 0.24 | 0.05 | 0.05 | 0.0003 | 0.0036 | 0.00014 | 0.008 | 0.0008 | 0.009 | 0.0012 |
| 2020.03 | M7 | 7.89 | 8.8 | 28 | 1.21 | 0.12 | 4.9 | 0.82 | 0.16 | 0.018 | 0.23 | 0.03 | 0.04 | 0.0003 | 0.0031 | 0.00012 | 0.008 | 0.0006 | 0.008 | 0.0009 |
| 2020.03 | M8 | 7.97 | 9.4 | 26 | 0.98 | 0.11 | 4.6 | 0.83 | 0.15 | 0.015 | 0.24 | 0.02 | 0.03 | 0.0003 | 0.0028 | 0.00009 | 0.007 | 0.0003 | 0.007 | 0.0006 |
| 2020.04 | M1 | 7.48 | 7.2 | 32 | 2.02 | 0.32 | 8.6 | 0.48 | 0.14 | 0.015 | 0.25 | 0.06 | 0.05 | 0.0002 | 0.0035 | 0.00014 | 0.014 | 0.0008 | 0.013 | 0.0013 |
| 2020.04 | M2 | 7.52 | 8.6 | 34 | 0.79 | 0.18 | 4.2 | 0.86 | 0.07 | 0.009 | 0.26 | 0.05 | 0.03 | 0.0002 | 0.0019 | 0.00008 | 0.007 | 0.0006 | 0.008 | 0.0008 |
| 2020.04 | T1 | 7.99 | 8.8 | 32 | 1.42 | 0.33 | 6.2 | 0.76 | 0.06 | 0.017 | 0.28 | 0.06 | 0.03 | 0.0002 | 0.0021 | 0.00007 | 0.013 | 0.0008 | 0.009 | 0.0009 |
| 2020.04 | T2 | 7.84 | 8.1 | 8 | 1.51 | 0.16 | 3.8 | 0.48 | 0.08 | 0.016 | 0.18 | 0.03 | 0.04 | 0.0003 | 0.0021 | 0.00008 | 0.008 | 0.0006 | 0.007 | 0.0009 |
| 2020.04 | T3 | 7.49 | 8.2 | 10 | 1.68 | 0.08 | 5.6 | 0.52 | 0.07 | 0.018 | 0.17 | 0.04 | 0.04 | 0.0002 | 0.0019 | 0.00016 | 0.008 | 0.0005 | 0.007 | 0.0007 |
| 2020.04 | M3 | 7.98 | 8.3 | 23 | 1.84 | 0.22 | 6.4 | 0.64 | 0.06 | 0.016 | 0.22 | 0.03 | 0.06 | 0.0003 | 0.0028 | 0.00012 | 0.009 | 0.0007 | 0.008 | 0.0013 |
| 2020.04 | M4 | 7.87 | 8.1 | 32 | 1.32 | 0.22 | 5.6 | 0.63 | 0.08 | 0.017 | 0.28 | 0.05 | 0.05 | 0.0004 | 0.0028 | 0.00014 | 0.013 | 0.0008 | 0.012 | 0.0016 |
| 2020.04 | T4 | 7.68 | 8.6 | 8 | 0.86 | 0.16 | 2.9 | 0.55 | 0.07 | 0.015 | 0.22 | 0.02 | 0.04 | 0.0004 | 0.0019 | 0.00009 | 0.009 | 0.0006 | 0.005 | 0.0008 |
| 2020.04 | T5 | 7.86 | 8.6 | 13 | 0.68 | 0.05 | 3.9 | 0.53 | 0.06 | 0.008 | 0.19 | 0.04 | 0.04 | 0.0002 | 0.0018 | 0.00008 | 0.007 | 0.0005 | 0.006 | 0.0008 |
| 2020.04 | M5 | 7.96 | 7.5 | 28 | 1.22 | 0.16 | 7.2 | 0.62 | 0.08 | 0.014 | 0.26 | 0.05 | 0.03 | 0.0003 | 0.0036 | 0.00013 | 0.011 | 0.0004 | 0.007 | 0.0014 |
| 2020.04 | M6 | 7.81 | 7.8 | 21 | 1.64 | 0.13 | 4.2 | 0.75 | 0.18 | 0.021 | 0.25 | 0.04 | 0.04 | 0.0003 | 0.0032 | 0.00013 | 0.009 | 0.0004 | 0.007 | 0.0009 |
| 2020.04 | T6 | 7.76 | 8.6 | 28 | 0.85 | 0.22 | 5.5 | 0.85 | 0.18 | 0.018 | 0.23 | 0.04 | 0.05 | 0.0003 | 0.0035 | 0.00014 | 0.008 | 0.0008 | 0.009 | 0.0013 |
| 2020.04 | M7 | 7.82 | 8.6 | 26 | 1.24 | 0.14 | 5.2 | 0.83 | 0.16 | 0.017 | 0.24 | 0.03 | 0.03 | 0.0003 | 0.0031 | 0.00011 | 0.009 | 0.0007 | 0.007 | 0.0009 |
| 2020.04 | M8 | 7.96 | 9.2 | 25 | 0.96 | 0.12 | 5.6 | 0.82 | 0.17 | 0.016 | 0.22 | 0.02 | 0.05 | 0.0003 | 0.0027 | 0.00009 | 0.007 | 0.0003 | 0.006 | 0.0008 |
| 2020.05 | M1 | 7.79 | 7.8 | 27 | 1.94 | 0.37 | 9.8 | 0.45 | 0.16 | 0.015 | 0.28 | 0.06 | 0.05 | 0.0002 | 0.0035 | 0.00013 | 0.014 | 0.0008 | 0.012 | 0.0006 |
| 2020.05 | M2 | 7.61 | 8.7 | 20 | 0.90 | 0.13 | 4.5 | 0.86 | 0.08 | 0.009 | 0.29 | 0.06 | 0.05 | 0.0002 | 0.0021 | 0.00009 | 0.007 | 0.0005 | 0.014 | 0.0007 |
| 2020.05 | T1 | 7.47 | 7.9 | 42 | 1.52 | 0.38 | 6.2 | 0.76 | 0.06 | 0.019 | 0.31 | 0.08 | 0.04 | 0.0002 | 0.0021 | 0.00007 | 0.011 | 0.0009 | 0.015 | 0.0009 |
| 2020.05 | T2 | 7.47 | 8.2 | 10 | 1.20 | 0.16 | 3.8 | 0.46 | 0.07 | 0.016 | 0.21 | 0.05 | 0.03 | 0.0003 | 0.0022 | 0.00009 | 0.007 | 0.0005 | 0.007 | 0.0005 |
| 2020.05 | T3 | 7.64 | 8.3 | 12 | 1.80 | 0.09 | 5.1 | 0.51 | 0.08 | 0.016 | 0.18 | 0.05 | 0.04 | 0.0002 | 0.0019 | 0.00018 | 0.008 | 0.0005 | 0.008 | 0.0003 |
| 2020.05 | M3 | 7.74 | 7.8 | 21 | 1.90 | 0.26 | 6.6 | 0.62 | 0.07 | 0.015 | 0.22 | 0.04 | 0.05 | 0.0003 | 0.0029 | 0.00013 | 0.008 | 0.0008 | 0.006 | 0.0005 |
| 2020.05 | M4 | 7.76 | 9.1 | 32 | 1.21 | 0.12 | 4.8 | 0.62 | 0.07 | 0.019 | 0.27 | 0.04 | 0.03 | 0.0004 | 0.0032 | 0.00014 | 0.012 | 0.0008 | 0.013 | 0.0005 |
| 2020.05 | T4 | 7.74 | 7.6 | 14 | 0.50 | 0.14 | 2.5 | 0.56 | 0.06 | 0.014 | 0.23 | 0.03 | 0.04 | 0.0004 | 0.0021 | 0.00009 | 0.009 | 0.0007 | 0.008 | 0.0006 |
| 2020.05 | T5 | 7.32 | 7.6 | 8 | 0.62 | 0.07 | 3.5 | 0.58 | 0.05 | 0.008 | 0.19 | 0.02 | 0.03 | 0.0002 | 0.0019 | 0.00008 | 0.008 | 0.0003 | 0.006 | 0.0005 |
| 2020.05 | M5 | 7.86 | 7.7 | 42 | 1.12 | 0.13 | 6.6 | 0.64 | 0.09 | 0.014 | 0.26 | 0.04 | 0.04 | 0.0003 | 0.0036 | 0.00014 | 0.012 | 0.0005 | 0.007 | 0.0007 |
| 2020.05 | M6 | 7.61 | 8.1 | 36 | 0.89 | 0.11 | 4.1 | 0.83 | 0.19 | 0.023 | 0.28 | 0.05 | 0.05 | 0.0004 | 0.0035 | 0.00013 | 0.008 | 0.0004 | 0.006 | 0.0007 |
| 2020.05 | T6 | 7.59 | 8.2 | 18 | 0.70 | 0.17 | 5.1 | 0.86 | 0.17 | 0.019 | 0.26 | 0.06 | 0.04 | 0.0003 | 0.0037 | 0.00015 | 0.007 | 0.0007 | 0.009 | 0.0008 |
| 2020.05 | M7 | 7.49 | 7.6 | 26 | 0.99 | 0.16 | 4.6 | 0.85 | 0.18 | 0.019 | 0.25 | 0.04 | 0.03 | 0.0003 | 0.0032 | 0.00013 | 0.008 | 0.0006 | 0.008 | 0.0006 |
| 2020.05 | M8 | 7.62 | 7.9 | 28 | 0.86 | 0.15 | 4.2 | 0.84 | 0.16 | 0.016 | 0.26 | 0.02 | 0.04 | 0.0003 | 0.0029 | 0.00008 | 0.008 | 0.0004 | 0.006 | 0.0006 |
| 2020.06 | M1 | 7.78 | 7.9 | 35 | 1.79 | 0.31 | 7.9 | 0.45 | 0.17 | 0.016 | 0.29 | 0.06 | 0.04 | 0.0003 | 0.0033 | 0.00026 | 0.018 | 0.0013 | 0.013 | 0.0006 |
| 2020.06 | M2 | 7.68 | 8.8 | 9 | 0.70 | 0.16 | 3.5 | 0.87 | 0.09 | 0.008 | 0.28 | 0.07 | 0.03 | 0.0003 | 0.0032 | 0.00025 | 0.017 | 0.0007 | 0.012 | 0.0008 |
| 2020.06 | T1 | 7.49 | 8.1 | 42 | 1.42 | 0.35 | 6.6 | 0.75 | 0.07 | 0.019 | 0.31 | 0.08 | 0.05 | 0.0003 | 0.0035 | 0.00026 | 0.015 | 0.0013 | 0.014 | 0.0007 |
| 2020.06 | T2 | 7.48 | 8.5 | 4 | 1.13 | 0.3 | 4.6 | 0.47 | 0.08 | 0.017 | 0.22 | 0.06 | 0.03 | 0.0003 | 0.0015 | 0.00009 | 0.009 | 0.0008 | 0.007 | 0.0006 |
| 2020.06 | T3 | 7.66 | 8.4 | 6 | 1.49 | 0.09 | 5.2 | 0.52 | 0.09 | 0.015 | 0.19 | 0.05 | 0.03 | 0.0002 | 0.0017 | 0.00013 | 0.008 | 0.0006 | 0.009 | 0.0004 |
| 2020.06 | M3 | 7.76 | 7.9 | 23 | 1.24 | 0.19 | 5.8 | 0.63 | 0.06 | 0.016 | 0.21 | 0.05 | 0.07 | 0.0003 | 0.0012 | 0.00015 | 0.008 | 0.0008 | 0.007 | 0.0005 |
| 2020.06 | M4 | 7.71 | 9.2 | 31 | 1.25 | 0.28 | 5.6 | 0.62 | 0.06 | 0.021 | 0.28 | 0.06 | 0.04 | 0.0002 | 0.0029 | 0.00016 | 0.007 | 0.0013 | 0.013 | 0.0006 |
| 2020.06 | T4 | 7.78 | 7.8 | 6 | 0.46 | 0.15 | 3.2 | 0.57 | 0.07 | 0.015 | 0.24 | 0.04 | 0.05 | 0.0003 | 0.0023 | 0.00015 | 0.012 | 0.0007 | 0.009 | 0.0006 |
| 2020.06 | T5 | 7.35 | 7.9 | 12 | 0.55 | 0.08 | 3.4 | 0.59 | 0.06 | 0.008 | 0.19 | 0.03 | 0.03 | 0.0002 | 0.0021 | 0.00015 | 0.011 | 0.0006 | 0.007 | 0.0007 |
| 2020.06 | M5 | 7.82 | 8.6 | 40 | 1.07 | 0.24 | 4.5 | 0.65 | 0.08 | 0.015 | 0.25 | 0.05 | 0.03 | 0.0003 | 0.0029 | 0.00018 | 0.019 | 0.0007 | 0.008 | 0.0006 |
| 2020.06 | M6 | 7.63 | 8.4 | 30 | 0.78 | 0.16 | 5.2 | 0.82 | 0.21 | 0.021 | 0.29 | 0.06 | 0.04 | 0.0005 | 0.0026 | 0.00016 | 0.018 | 0.0012 | 0.007 | 0.0006 |
| 2020.06 | T6 | 7.62 | 8.5 | 18 | 0.70 | 0.17 | 5.8 | 0.86 | 0.18 | 0.021 | 0.27 | 0.07 | 0.05 | 0.0004 | 0.0031 | 0.00018 | 0.009 | 0.0009 | 0.008 | 0.0007 |
| 2020.06 | M7 | 7.52 | 7.6 | 29 | 0.99 | 0.19 | 5.9 | 0.86 | 0.19 | 0.019 | 0.26 | 0.05 | 0.04 | 0.0004 | 0.0028 | 0.00016 | 0.016 | 0.0012 | 0.009 | 0.0008 |
| 2020.06 | M8 | 7.65 | 7.8 | 28 | 0.87 | 0.18 | 6.4 | 0.85 | 0.17 | 0.017 | 0.25 | 0.03 | 0.03 | 0.0003 | 0.0026 | 0.00015 | 0.012 | 0.0009 | 0.007 | 0.0006 |
| 2020.07 | M1 | 7.79 | 8.5 | 29 | 1.49 | 0.31 | 8.1 | 0.46 | 0.18 | 0.017 | 0.28 | 0.06 | 0.05 | 0.0003 | 0.0032 | 0.00026 | 0.019 | 0.0014 | 0.014 | 0.0006 |
| 2020.07 | M2 | 7.69 | 8.9 | 13 | 0.62 | 0.19 | 3.6 | 0.82 | 0.09 | 0.009 | 0.29 | 0.08 | 0.04 | 0.0003 | 0.0034 | 0.00024 | 0.018 | 0.0008 | 0.011 | 0.0008 |
| 2020.07 | T1 | 7.59 | 8.5 | 16 | 1.38 | 0.2 | 6.7 | 0.76 | 0.08 | 0.018 | 0.32 | 0.07 | 0.03 | 0.0003 | 0.0036 | 0.00025 | 0.016 | 0.0014 | 0.015 | 0.0007 |
| 2020.07 | T2 | 7.51 | 8.9 | 9 | 1.82 | 0.29 | 4.8 | 0.46 | 0.09 | 0.019 | 0.23 | 0.06 | 0.04 | 0.0003 | 0.0015 | 0.00011 | 0.008 | 0.0009 | 0.008 | 0.0006 |
| 2020.07 | T3 | 7.69 | 8.8 | 8 | 1.52 | 0.08 | 5.5 | 0.51 | 0.08 | 0.015 | 0.21 | 0.05 | 0.04 | 0.0003 | 0.0018 | 0.00012 | 0.009 | 0.0007 | 0.008 | 0.0004 |
| 2020.07 | M3 | 7.86 | 8.2 | 13 | 0.87 | 0.18 | 5.9 | 0.62 | 0.07 | 0.016 | 0.23 | 0.06 | 0.03 | 0.0003 | 0.0015 | 0.00016 | 0.007 | 0.0007 | 0.009 | 0.0005 |
| 2020.07 | M4 | 7.74 | 9.4 | 28 | 1.52 | 0.28 | 5.8 | 0.61 | 0.07 | 0.022 | 0.25 | 0.05 | 0.03 | 0.0002 | 0.0031 | 0.00015 | 0.006 | 0.0014 | 0.011 | 0.0006 |
| 2020.07 | T4 | 7.82 | 7.9 | 19 | 0.46 | 0.17 | 3.5 | 0.58 | 0.06 | 0.016 | 0.24 | 0.03 | 0.04 | 0.0003 | 0.0024 | 0.00016 | 0.013 | 0.0008 | 0.009 | 0.0006 |
| 2020.07 | T5 | 7.42 | 8.5 | 9 | 0.87 | 0.15 | 3.6 | 0.56 | 0.06 | 0.009 | 0.21 | 0.04 | 0.05 | 0.0002 | 0.0023 | 0.00014 | 0.013 | 0.0007 | 0.008 | 0.0007 |
| 2020.07 | M5 | 7.91 | 8.9 | 36 | 1.12 | 0.26 | 4.8 | 0.62 | 0.08 | 0.015 | 0.23 | 0.03 | 0.03 | 0.0002 | 0.0028 | 0.00019 | 0.018 | 0.0006 | 0.009 | 0.0006 |
| 2020.07 | M6 | 7.82 | 8.6 | 27 | 0.73 | 0.15 | 5.4 | 0.81 | 0.19 | 0.022 | 0.28 | 0.06 | 0.04 | 0.0004 | 0.0024 | 0.00015 | 0.017 | 0.0013 | 0.007 | 0.0006 |
| 2020.07 | T6 | 7.71 | 8.6 | 17 | 0.86 | 0.21 | 5.9 | 0.82 | 0.18 | 0.021 | 0.26 | 0.08 | 0.03 | 0.0004 | 0.0032 | 0.00017 | 0.008 | 0.0008 | 0.009 | 0.0007 |
| 2020.07 | M7 | 7.53 | 7.9 | 29 | 0.82 | 0.19 | 6.1 | 0.82 | 0.19 | 0.021 | 0.24 | 0.06 | 0.04 | 0.0004 | 0.0027 | 0.00015 | 0.017 | 0.0013 | 0.008 | 0.0008 |
| 2020.07 | M8 | 7.68 | 8.2 | 26 | 0.73 | 0.17 | 6.6 | 0.83 | 0.18 | 0.018 | 0.25 | 0.04 | 0.03 | 0.0003 | 0.0025 | 0.00018 | 0.013 | 0.0008 | 0.006 | 0.0006 |
