# Supplementary material for: Water quality assessment and source identification of the Shuangji River (China) using multivariate statistical methods
Source: PLoS One. 2021 Jan 22;16(1):e0245525. doi: 10.1371/journal.pone.0245525 (PMC7822302; doi:10.1371/journal.pone.0245525)
Supplement: Attachment — Submitted filename: Response to reviewers.docx [file pone.0245525.s002.docx]

**Dear Editors and Reviewers,**

We highly appreciate the valuable comments of the editors and reviewers on our manuscript of PONE-D-20-20664'. The suggestions are quite helpful for us and we have incorporated them into the revised paper. We have checked the manuscript carefully, and revised it according to the comments of the editors and reviewers to improve its quality. As below, specific response to the comments were provided point by point. The revised sentences of the manuscript were highlighted in red in revised manuscript. We hope the editors and the reviewers will be satisfied with our responses to the comments and the revisions for the original manuscript.

Thank you very much.

Sincerely yours,

Dong Zhang

**Reply to reviewer(s)' Comments:**

**Reviewer #1:**

Comments:

The specific comments on the first review of your manuscript "Water quality assessment and source identification of the Shuangji River (China) using multivariate statistical methods" (PONE-D-20-20664) are as follows:

1. I highly recommend authors to improve the language quality.

**Response:** First of all, we would like to express our gratitude for your time-consuming review of our manuscript. According to your valuable comments, we have invited expert to revise our manuscript. We hope the reviewers and editors will be satisfied with the revisions for the original manuscript. Related revisions for the manuscript were made in red color according to actual circumstances and located in page 1 (lines 5, 6, 13, and 18), page 5 (lines 97-99 and 110), page 6 (lines 111-112, and 114), page 7 (lines 133, 136, 146 and 151-152), page 8 (lines 158-169 and 172-174), page 9 (lines 175-178 and 181), page 10 (line 191), page 11 (line 206), page 12 (line 207), page 13 (lines 211 and 228), page 14 (lines 233,238 and 250 ), page 17 (lines 293 and 296), page 18 (lines 311-312 and 321), page 19 (lines 329, 331 and 332), page 20 (line 343), page 21 (line 352), page 22 (lines 360 and 365), page 23 (lines 368 and 375), page 24 (lines 379 and 389), page 25 (line 393), page 26 (lines 404-405, and 407-408), page 27 (lines 420, 424 and 431), page 28 (lines 433-436 and 441), page 29 (lines 444-445, 450, 454 and 456), page 30 (lines 474,478 and 481), page 31 (line 493).

2. Introduction should be re-written as it is hard to know the main insight of the paper. And key statistical methods are not well presented.

**Response:** Thank you very much for your valuable suggestion. Based on your comments, we have rewritten the introduction and added an introduction to statistical methods in the introduction. Meanwhile, the related description has been provided in the text in pages 3-5, lines 45-91 in red.

Introduction

Water is the material basis for the existence of earth creatures, and water resources are the primary condition for maintaining the sustainable development of the earth's ecological environment [1].With the increasing consumption of water resources, the contradiction between the supply and demand of water resources has intensified, which puts forward greater requirements for the utilization and protection of surface water resources [2].

The surface water quality of a region depends to a large extent on envirnmental factors (temperature changes, precipitation and soil erosion) and and human input (discharge of municipal and industrial wastewater and over-exploitation of water resources) [3]. Among them, the discharge of urban sewage and industrial wastewater is a continuous source of pollution, so effective control of sewage discharge is of great significance to the improvement of water quality [4, 5].

Surface water runoff is a seasonal phenomenon that is mainly affected by the climate of the basin [6]. In addition, seasonal changes in precipitation, surface runoff, interflow, groundwater flow, and pumping in and pumping out have a strong influence on the river flow and the subsequent pollutant concentration in the river [7].Therefore, correct identification of potential sources of surface water quality pollution is the basis and prerequisite for water quality management.

Shuangji River is a polluted river. Its main source of water comes from urban sewage treatment plants and paper-making sewage treatment plants. It not only plays an important role in assimilating or removing urban and industrial wastewater and farmland runoff, but is also the the main inland water resources used for household, industrial, and irrigation purposes [8], Therefore, it is necessary to prevent and control river pollution and have reliable water quality information for effective management. Given the spatial and temporal changes in river water chemistry, regular monitoring programmes are needed to reliably estimate water quality [9]. This leads large and complex data matrices composed of a large number of physical and chemical parameters, which are often difficult to interpret, making it challenging to draw meaningful conclusions [9].

Multivariate statistical analysis is a branch developed from classical statistics and is a comprehensive analysis method [11, 12]. It can analyze the statistical laws of multiple objects and multiple indicators when they are related to each other, including cluster analysis (CA) [13], discriminant analysis (DA) [14], principal component analysis (PCA) [15] and factor analysis (FA) [16]. Multivariate statistical analysis is a suitable tool for multi-component chemical and physical measurements for meaningful data reduction and interpretation [17]. It is a valuable tool for identifying factors and sources that may affect water systems and cause changes in water quality [18].

In this article, we took the Shuangji River as the research object for the first time, set up 14 main detection points along the river and detected and analyzed 19 physical and chemical parameters in water samples. The detection time lasted for 2 years.Different multivariate statistical techniques were used to analyse the obtained datasets, to analyse the similarity or dissimilarity between monitoring periods or monitoring points, to identify the water quality variables that cause the spatial and temporal changes of river water quality, and to determine the impact of water sources (natural and anthropogenic factors).

3. Line 78, The author should unify the accuracy of the values.

**Response:** Thank you very much for your valuable suggestion. According to your suggestions, we have checked 78 lines of sentences and confirmed their accuracy, In addition, we have carefully checked the whole manuscript to avoid similar mistakes elsewhere.

4. The format of the remarks in Table 1 needs to be unified with the format in the table.

**Response:** Thanks for the reviewer’s valuable advice. The problem pointed out by the reviewers has been checked according to your advice and the related information can be seen in page 9, line181.

Table 1 is as follows

Table 1. Water quality parameters associated with their acronyms, units and analytical methods used

| **S.N.** | **Variables** | **Acronyms** | **Units** | **Analytical methods** |
| --- | --- | --- | --- | --- |
| **1** | pH | pH | pH unit | pH meter |
| **2** | Dissolved oxygen | DO | mg/L | Prob metod |
| **3** | Chemical oxygen demand | COD | mg/L | Dichromate method |
| **4** | Ammonical nitrogen | NH3-N | mg/L | Spectrophotometric |
| **5** | Total phosphorus | TP | mg/L | Ammonium molybdate Spectrophotometry |
| **6** | Chemical oxygen demand(Mn) | CODMn | mg/L | Permanganate index method |
| **7** | Fluoride | F | mg/L | Ion selective electrode |
| **8** | Petroleum oil | Oil | mg/L | Infrared spectrophotometry |
| **9** | Linear alkylbenzene sulfonates | LAS | mg/L | Methylene blue Spectrophotometry |
| **10** | Copper | Cu | mg/L | FAAS |
| **11** | Zinc | Zn | mg/L | FAAS |
| **12** | Cadmium | Cd | mg/L | ETAAS |
| **13** | Arsenic | As | mg/L | HGAAS |
| **14** | Mercury | Hg | mg/L | CVAAS |
| **15** | Hexavalent chromium | Cr6+ | mg/L | ETAAS |
| **16** | Total cyanide | CN | mg/L | Pyridine barbituric acid Spectrophotometry |
| **17** | Volatile phenol | VP | mg/L | Spectrophotometric Determination with 4-Amino-Antipyrin |
| **18** | Sulfide | S | mg/L | Methylene blue Spectrophotometry |
| **19** | Selenium | Se | mg/L | HGAAS |

5. The accuracy of the values in Table 3 should be unified.

**Response:** Thanks for the reviewer’s valuable advice. The Table pointed out by the reviewers has been checked according to your advice and confirmed their accuracy, In addition, we have carefully checked the whole manuscript to avoid similar mistakes elsewhere.

Table 3 is as follows

Table 3. Results of temporal-DA for temporal variation

| **Modes** | **DF** | **R** | **Wilks’lambda** | **Chi-square** | **P-level** |
| --- | --- | --- | --- | --- | --- |
| **Standard** | 1 | 0.728 | 0.273 | 202.505 | 0.000 |
|  | 2 | 0.647 | 0.581 | 84.734 | 0.000 |
| **Forward** | 1 | 0.726 | 0.279 | 201.718 | 0.000 |
|  | 2 | 0.640 | 0.590 | 83.276 | 0.000 |
| **Backward** | 1 | 0.659 | 0.457 | 128.057 | 0.000 |
|  | 2 | 0.438 | 0.808 | 34.834 | 0.000 |

6. Line 251-256 The Author need to add relevant literature as supplementary proof.

**Response:** Thank you very much for your valuable suggestion. According to your valuable advice, we selected three references, related references were listed as follows. The related description has been provided in the text in page 15, lines 259, 264 and 268, and References in red (in pages 37-38, lines 631-643).

[45]	González MJG, Vallejo-Pascual M. The Application of Principal Component Analysis (PCA) for The Study of The Spanish Tourist Demand. Quaestiones geographicae. 2018 2018-01-01;37(4):43-52.

[46]	Mohamad Asri MN, Mat Desa WNS, Ismail D. Combined Principal Component Analysis (PCA) and Hierarchical Cluster Analysis (HCA): an efficient chemometric approach in aged gel inks discrimination. AUST J FORENSIC SCI. 2020 2020-01-02;52(1):38-59.

[48]	Ding S, Jia W, Su C, Zhang L, Liu L. Research of neural network algorithm based on factor analysis and cluster analysis. Neural Computing and Applications. 2011;20(2):297-302.

7. A table with higher pollution intensity needs to be added to as the support information.

**Response:** Thanks for the comment of the review. The issues pointed out by the reviewers has been added according to your advice.

The table is as follows

Table 2-1 High pollution intensity in water bodies

| **Parameters** |  | **M1** | **M2** | **Class III** | **Class IV** |
| --- | --- | --- | --- | --- | --- |
| **pH** | Mean | 7.56 | 7.63 | 6-9 | 6-9 |
| **DO (mg/L)** | Mean | 7.57 | 8.56 | 5 | 3 |
| **COD (mg/L)** | Mean | 30.08 | 26.92 | 20 | 30 |
| **NH3-N (mg/L)** | Mean | 1.64 | 1.39 | 1.0 | 1.5 |
| **TP (mg/L)** | Mean | 0.38 | 0.31 | 0.05 | 0.1 |
| **CODMn (mg/L)** | Mean | 8.28 | 7.09 | 6 | 10 |
| **F (mg/L)** | Mean | 0.55 | 0.84 | 1.0 | 1.5 |
| **Oil (mg/L)** | Mean | 0.17 | 0.11 | 0.05 | 0.5 |
| **Cr6+ (mg/L)** | Mean | 0.018 | 0.019 | 0.05 | 0.05 |
| **LAS (mg/L)** | Mean | 0.27 | 0.24 |  |  |
| **Cu (mg/L)** | Mean | 0.06 | 0.07 | 1.0 | 1.0 |
| **Zn (mg/L)** | Mean | 0.05 | 0.04 | 1.0 | 2.0 |
| **Cd (mg/L)** | Mean | 0.00024 | 0.00027 | 0.05 | 0.1 |
| **As (mg/L)** | Mean | 0.0029 | 0.0025 | 0.05 | 0.1 |
| **Hg (mg/L)** | Mean | 0.00017 | 0.00015 | 0.0001 | 0.0005 |
| **CN (mg/L)** | Mean | 0.015 | 0.013 | 0.2 | 0.2 |
| **VP (mg/L)** | Mean | 0.0011 | 0.0010 | 0.005 | 0.1 |
| **S (mg/L)** | Mean | 0.012 | 0.012 | 0.2 | 0.5 |
| **Se (mg/L)** | Mean | 0.0009 | 0.0009 | 0.01 | 0.02 |

8. Line 290, it needs better reasons to divide pollution levels.

**Response:** Thank you very much for your careful review and the valuable suggestion. According to your opinion, we have added the reasons for the classification of pollution levels, and the related revision can be seen in page 18, lines 311-312.

9. The format of the ordinate of Figures 2, 4, 5 and 6 needs to be unified, and spaces need to be added before the brackets. The format is the new Roman.

**Response:** Thanks for the comment of the review. According to your valuable comments, we have checked the errors and revised them. Detailed information about the places where revisions was displayed in page 17, line 293, page 22, line 360 , page 25, line 393, page 27, line 420. In addition, we have carefully checked the whole manuscript to avoid similar mistakes elsewhere (page 18, line 321)

The revised Fig. 2, Fig. 4, Fig. 5, Fig. 6 were shown as follows.

**Fig 2. Dendrogram showing temporal clustering of monitoring periods**

**Fig 4. The (a)- (d) temporal variations: CODMn, Cu, As and Se**

**Fig 5.The (a)-(g) spatial variations: COD, TP, CODMn, F, LAS, Cu and Cd**

**Fig 6. Scree Plot of variance of PCs**

The revised Fig 3 was shown as follows

**Fig 3. Dendrogram showing spatial clustering of monitoring sites**

10. Some minor typos and inappropriate use of words should be cleaned up.

**Response:** Thanks for the comment of the review. According to your valuable comments, we have checked the errors and revised them. Detailed information about the places where revisions were made in red color was displayed in page 1 (lines 5, 6, 13, and 18), page 5 (lines 97-99 and 110), page 6 (lines 111-112, and 114), page 7 (lines 133, 136, 146 and 151-152), page 8 (lines 158-169 and 172-174), page 9 (lines 175-178 and 181), page 10 (line 191), page 11 (line 206), page 12 (line 207), page 13 (lines 211 and 228), page 14 (lines 233,238 and 250 ), page 17 (lines 293 and 296), page 18 (lines 311-312 and 321), page 19 (lines 329, 331 and 332), page 20 (line 343), page 21 (line 352), page 22 (lines 360 and 365), page 23 (lines 368 and 375), page 24 (lines 379 and 389), page 25 (line 393), page 26 (lines 404-405, and 407-408), page 27 (lines 420, 424 and 431), page 28 (lines 433-436 and 441), page 29 (lines 444-445, 450, 454 and 456), page 30 (lines 474,478 and 481), page 31 (line 493). Besides, we have invited expert to revise our manuscript. And some doctoral students in our research group have also tried our best to improve the language in the revised manuscript. We hope the reviewers and editors will be satisfied with the revisions for the original manuscript.

11. Line 398-401, the expression is obscure.

**Response:** Thank you very much for your valuable suggestion. We have checked this sentence carefully, it means that previous experimental studies have shown that when the characteristic value of the principal component is greater than 1, most of the factor variables can be expressed. It can be seen from Figure 6 that in the sixth principal component, the eigenvalue is greater than 1, that is, 19 factor variables can be expressed.

12. The meaning of the topic needs to be further clarified and expanded, and the corresponding preface and discussion need to be further improved. Currently, the paper lacks discussion based on results (various correlations) and cannot be limited to the analysis of data values. It is necessary to use software such as spass to analyze the correlation of data. These aspects require the author to refer to the latest research progress at home and abroad, condense the scientific issues, and then start a systematic discussion around the basic results of this article. Secondly, the corresponding preface must be reorganized.

**Response:** Many thanks for the reviewer’s affirmation to our manuscript. According to your suggestion, we have increased the discussion of the results. Detailed information about the places where revisions were made in red color was displayed in page 16, lines 284-292, page 26, lines 410-414, page 28, lines 437-440, page 29, lines 442-453, pages 29 and 30, lines 459-465. The introduction has also been revised, and the relevant revisions are in pages 3, 4 and 5, lines 45-91.

Introduction

Water is the material basis for the existence of earth creatures, and water resources are the primary condition for maintaining the sustainable development of the earth's ecological environment [1].With the increasing consumption of water resources, the contradiction between the supply and demand of water resources has intensified, which puts forward greater requirements for the utilization and protection of surface water resources [2].

The surface water quality of a region depends to a large extent on envirnmental factors (temperature changes, precipitation and soil erosion) and and human input (discharge of municipal and industrial wastewater and over-exploitation of water resources) [3]. Among them, the discharge of urban sewage and industrial wastewater is a continuous source of pollution, so effective control of sewage discharge is of great significance to the improvement of water quality [4, 5].

Surface water runoff is a seasonal phenomenon that is mainly affected by the climate of the basin [6]. In addition, seasonal changes in precipitation, surface runoff, interflow, groundwater flow, and pumping in and pumping out have a strong influence on the river flow and the subsequent pollutant concentration in the river [7].Therefore, correct identification of potential sources of surface water quality pollution is the basis and prerequisite for water quality management.

Shuangji River is a polluted river. Its main source of water comes from urban sewage treatment plants and paper-making sewage treatment plants. It not only plays an important role in assimilating or removing urban and industrial wastewater and farmland runoff, but is also the the main inland water resources used for household, industrial, and irrigation purposes [8], Therefore, it is necessary to prevent and control river pollution and have reliable water quality information for effective management. Given the spatial and temporal changes in river water chemistry, regular monitoring programmes are needed to reliably estimate water quality [9]. This leads large and complex data matrices composed of a large number of physical and chemical parameters, which are often difficult to interpret, making it challenging to draw meaningful conclusions [9].

Multivariate statistical analysis is a branch developed from classical statistics and is a comprehensive analysis method [11, 12]. It can analyze the statistical laws of multiple objects and multiple indicators when they are related to each other, including cluster analysis (CA) [13], discriminant analysis (DA) [14], principal component analysis (PCA) [15] and factor analysis (FA) [16]. Multivariate statistical analysis is a suitable tool for multi-component chemical and physical measurements for meaningful data reduction and interpretation [17]. It is a valuable tool for identifying factors and sources that may affect water systems and cause changes in water quality [18].

In this article, we took the Shuangji River as the research object for the first time, set up 14 main detection points along the river and detected and analyzed 19 physical and chemical parameters in water samples. The detection time lasted for 2 years.Different multivariate statistical techniques were used to analyse the obtained datasets, to analyse the similarity or dissimilarity between monitoring periods or monitoring points, to identify the water quality variables that cause the spatial and temporal changes of river water quality, and to determine the impact of water sources (natural and anthropogenic factors).

13. The reference section has a format error or lacks relevant information, such as: Reference 2, please carefully follow the submission requirements.

**Response:** Thanks for the comment of the review. According to your valuable comments, we have checked the errors and revised them. Detailed information about the places where revisions were made in red color was displayed reference. In addition, we have carefully checked the whole manuscript to avoid similar mistakes elsewhere.

14. The authors only presented the results achieved, without really discussing them in a deeper discussion.

**Response:** Thanks for your valuable comments. According to your suggestion, we have increased the discussion of the results, and the relevant information was also provided in the revised manuscript (page 16, lines 284-292, page 26, lines 410-414, page 28, lines 437-440, page 29, lines 442-453, pages 29 and 30, lines 459-465). Since the Shuangji River is mainly a polluted river, the main body of the river comes from the sewage treatment plant along the bank, and the change in water quality reflects the change in the treatment effect of the sewage treatment plant. In summer, the sewage treatment plant has a better treatment effect, and the summer rainfall is large, and the river flow is large, so the river water quality in summer is better and divided into one category. In winter, the sewage treatment plant has poor water quality due to temperature and operation, and the rainfall is small, and the river flow is small. Therefore, the river water quality in winter is poor and divided into one category.

FA/PCA analysis is aimed at the standardized data and compares and analyses the composition patterns between water samples to determine the important factors that affect each water sample [50]. The PCA of all datasets yielded six (principal component)PCs, which explained 68% of the total variance with eigenvalues > 1 (Table 1). The first PC (29.7% of the total variance) was correlated (loading >0.7) with COD, TP, Cu and VP. The third PC (9.2% of total variance) was correlated (loading>0.7) with LAS. However, the second, fourth, fifth and sixth PCs, although they accounted for the total variance of 10.4%, 7.2%, 5.9% and 5.5%, respectively, were not correlated (loading>0.7) with any of the parameters. Combining the local industrial structure and distribution, it characterizes emissions related to industrial industries such as handmade paper manufacturing, coking, chemical raw materials and chemical products manufacturing, and metal products, which are consistent with the current main industrial industries in Xinmi (Henan Province).

VF1 (17.7% of the total variance) had strong positive loadings on Cu and S and moderate positive loadings on NH3-N and TP, indicating pollution from mineral composition and domestic sewage. This is because the main source of rivers in Xinmi City is drainage from domestic sewage plants, and the main source of pollution is domestic pollution sources, and the upstream of the tributaries are mainly coal and metal industries, causing some heavy metal pollution. VF2 (12.6% of total variance) had strong positive loadings on Cd and moderate positive loadings on Se and COD, indicating that the source was industrial wastewater pollution. This is related to the main paper industry, metal products, and steel casting manufacturing industries along the Shuangji River. VF3 (10.7% of total variance) had strong positive loadings on F and Oil and moderate positive loadings on Cr6+, mainly manifested as fluoride pollution and heavy metal pollution. This clustering indicates that the source of pollution was the discharge of wastewater from the chemical industry, which is related to some chemical industries and metal manufacturing industries along the river. VF4, which explained 9.9% of the total variance, had moderate positive loadings on CODMn, NH3-N and Zn, indicating that the pollution was from mineral-related hydrochemistry and domestic sewage, mainly from the discharge of wastewater from paper-making enterprises, domestic sewage, and metal manufacturing wastewater into the river along the coast. VF5 (8.9% of total variance) had moderate positive loadings on pH and LAS, which can be interpreted as coming from detergents and personal necessities in domestic sewage. VF6, which explained 7.9% of the total variance, had moderate negative loadings on DO. This finding suggests that the pollutants in the water consumed a large amount of oxygen.

The FA/PCA results indicated that most changes were composed of soluble salts (natural) and organic pollutants (artificial). FA/PCA indicated that the main pollutants are COD, CODMn, NH3-N, TP, Cu,Cr6+, Zn, S, Se, Cd, F, Oil and LAS. These pollutants mainly come from domestic sewage discharge COD, CODMn, NH3-N, TP; papermaking wastewater discharge COD, CODMn; textile industry, chemical product manufacturing wastewater discharge F, Oil and LAS; metal product manufacturing, optoelectronic device manufacturing and other industrial wastewater discharge Cu, Cr6+, Zn, S, Se and Cd. The FA can identify the parameters that have the greatest contributions to changes in river water quality. The method of assessing the spatiotemporal changes in water quality based on FA/PCA have been applied to water quality evaluation at an early stage.

15. The relationship between major influencing factors and pollution sources is not given , and the universality of statistical methods is questionable.

**Response:** Thanks for the reviewer’s valuable advice. The problem pointed out by the reviewers has been added according to your advice and the related information can be seen in discussion (page 16, lines 284-292, page 26, lines 410-414, page 28, lines 437-440, page 29, lines 442-453, pages 29 and 30, lines 459-465).

**Reviewer #2:**

Comments:

A large number of observation and analysis has been done by the authors, and this manuscript has some extent scientific significance. However, However, the submission is under standard at its present stage. A major revision is recommened.

1. What is the scientific significance of your study? Why readers should pay attention to your research?

**Response:** Many thanks for the reviewer’s affirmation to our manuscript. The scientific significance of this paper is to provide a basis for water quality analysis of polluted rivers.This is the first time that statistical analysis has been used to systematically evaluate the water quality of the Shuangji River and analyze the sources of pollutants in the Shuangji River. Therefore, this article can increase readers' interest.

2. Why the Shuangji River basin is chosen as the study area? Does it take more advantage over other regions? If yes, what these merits are?

**Response:** Thank you very much for your careful review and the valuable suggestion. The Shuangji River is an important river in Xinmi City. It carries the drainage of important sewage treatment plants in Xinmi City and the drainage of coastal enterprises. Its water sources are very complex, including paper industry water, domestic water, chemical industry water, and coal mine enterprise water. The complex water sources lead to the complexity of the water quality of the Shuangji River. If the water quality of the Shuangji River can be analyzed using scientific methods to simplify the complex water quality, this will be an example of solving complicated river pollution through scientific means. To provide a reference for the governance of other complex rivers, but also to provide scientific and effective methods for the work of the local government.

3. Language needs further improvement.

**Response:** Thanks for the comment of the review. According to your valuable comments, we have checked the errors and revised them. Detailed information about the places where revisions were made in red color was displayed in page 1 (lines 5, 6, 13, and 18), page 5 (lines 97-99 and 110), page 6 (lines 111-112, and 114), page 7 (lines 133, 136, 146 and 151-152), page 8 (lines 158-169 and 172-174), page 9 (lines 175-178 and 181), page 10 (line 191), page 11 (line 206), page 12 (line 207), page 13 (lines 211 and 228), page 14 (lines 233,238 and 250 ), page 17 (lines 293 and 296), page 18 (lines 311-312 and 321), page 19 (lines 329, 331 and 332), page 20 (line 343), page 21 (line 352), page 22 (lines 360 and 365), page 23 (lines 368 and 375), page 24 (lines 379 and 389), page 25 (line 393), page 26 (lines 404-405, and 407-408), page 27 (lines 420, 424 and 431), page 28 (lines 433-436 and 441), page 29 (lines 444-445, 450, 454 and 456), page 30 (lines 474,478 and 481), page 31 (line 493). Besides, we have invited expert to revise our manuscript. And some doctoral students in our research group have also tried our best to improve the language in the revised manuscript. We hope the reviewers and editors will be satisfied with the revisions for the original manuscript.

**Reviewer #3:**

Comments:

Water quality assessment and source identification of the Shuangji River (China) using multivariate statistical methods.
1. Assessment of river water quality using multivariate statistical tool help to identify the sources and parameters that lead to water pollution and largely changes due to this pollution load. The authors highlighted some physicochemical and trace metals that indicate pollution in the Shuangji river. Through the authors claim this is the first study of water quality assessment of the Shuangji river, the methods of assessment are not novel. Several studies (some of them cited by authors itself) used multivariate statistical techniques thus it lacks novelty. The authors must clearly explain the novelty of his work. Also, I do not find the research gap which motivates to conduct this study.

**Response:** First of all, we would like to express our gratitude for your time-consuming review of our manuscript.The scientific significance of this paper is to provide a basis for water quality analysis of polluted rivers.This is the first time that statistical analysis has been used to systematically evaluate the water quality of the Shuangji River and analyze the sources of pollutants in the Shuangji River. Therefore, this article can increase readers' interest.

2. Line 23-27- it is a well-established fact. Many of us did this already.

L 57 it is not a natural process, they are environmental factors

L 87 Study reported data for 2 yrs i.e. 2019 and 2020. How? 2020 is still going on. The authors also mentioned the Covid-19 problem in data collection. It looks the data is only for one year. Please correct.

L 95 mention N and E in geographical coordinates

L 463 – 465 again a well-known fact. Please provides some novel fining and scope of the study. What other researches should learn from your work. What is the future implementation of your work? Please make it more appealing.

Table 2 makes it more compact. Like in one row provide mean ± SD and min-max in the second row.

Fig. 6 transfer it in supplementary figures

**Response:** Thanks for the comment of the review. According to your valuable comments, we have checked the errors and revised them. Detailed information about the places where revisions were made in red color was displayed in page 3, lines 52 and 53.

The data of the study is one years (2019-2020). We have revised the time of the article. The relevant information is in page 1, line 3.

The geographic information of the Xinmi city has also been revised (page 5, line 94).

The Shuangji River is an important river in Xinmi City. It carries the drainage of important sewage treatment plants in Xinmi City and the drainage of coastal enterprises. Its water sources are very complex, including paper industry water, domestic water, chemical industry water, and coal mine enterprise water. The complex water sources lead to the complexity of the water quality of the Shuangji River. If the water quality of the Shuangji River can be analyzed using scientific methods to simplify the complex water quality, this will be an example of solving complicated river pollution through scientific means. To provide a reference for the governance of other complex rivers, but also to provide scientific and effective methods for the work of the local government.

We tried to modify Table 2 according to your suggestion, but the width of the modified table exceeds the layout, so that the table is divided into two, which affects the reading, so we boldly keep the original format.

3. The authors should provide details of the instruments used for analysis. Model and make of instruments. What is the analytical performance of the various instruments? Details of LOD, SRM is missing. Provide the relevance of highly altered parameters with respect to human health, the scope of water treatment before discharge.

**Response:** Thanks for the comment of the review. Based on your opinion, we have provided detailed information on the equipment used for analysis. Detailed information about the places where revisions were made in red color was displayed in page 7, lines 158-169 and 174, page 9, lines 175-178.

The pH value and DO value of each water sample were determined on site using digital pH values (JY-PH6.0) and DO measuring instruments (YT-RJY). The COD was measured by the dichromate reflux method (DH310C1COD) [20], and NH3-N was measured with Nessler’s reagent (NH_3_N-1040) [21]. The TP was measured by ammonium molybdate spectrophotometry (HM-812) [22], and the CODMn was measured by the permanganate index method (Thermo Scientific 3131) [22]. Fluoride (F) was measured using an ion-selective electrode ([BHF5300](http://www.bellchina.com.cn/prod_show.aspx?cid=211&id=655)) [24], and the total cyanides were analysed using pyridine barbituric acid spectrophotometry (TCN-508) [25], while sulphide (S) was determined using methylene blue spectrophotometry (ST201A) [26]. Petroleum hydrocarbons (Oil) were measured using infrared spectrophotometry (GC1290) [27]. The linear alkylbenzene sulfonates (LAS) were measured using methylene blue spectrophotometry (UltiMate3000) [28], and volatile phenols (VP) were measured using spectrophotometric determination with 4-amino-antipyrin (BELL) [29].

The main cation was determined by subjecting the acid-treated water samples to a 20-fold dilution with ultrapure water. For the trace elements and toxic elements, the volume of the water samples was reduced by a factor of four at 60 ℃ on an electric hot plate. Cu and Zn were determined by a flame atomic absorption spectrometer (FAAS) using an ethane-air flame (CAAM-2001N) [29], while Hg was determined by cold-vapor atomic absorption spectrometry (CVAAS) (Ultima Expert) [31]. Cd and Cr were measured using an electrothermal atomic absorption spectrometer (ETAAS) (Avio 200) [32], while As and Se were analysed using the hydride generation method (HGAAS) (AA-6033C)[33]. The accuracy of the analytical data were ensured by triplicate samples, blank test controls and careful standardization. The ion balance of each sample was within ±5%.
